# Supplementary material for: Treatment Pathways and Prognosis in Advanced Sarcoma with Peritoneal Sarcomatosis
Source: Cancers (Basel). 2023 Feb 20;15(4):1340. doi: 10.3390/cancers15041340 (PMC9954769; doi:10.3390/cancers15041340)
Supplement: Supplementary file 1 [file cancers-15-01340-s001.zip › cancers-2194625-supplementary.pdf]

Table S1. Detailed patient characteristics

Patient	Age	Sex	Subtype	Primary origin	Time to PS in months	Number of resections	Intention	Additional therapy	Complications	survival after PS in months
**1**	67	m	NOS	retropubic space	20	7	curative	CTX	impaired wound healing	21
**2**	81	f	NOS	retroperitoneal space	63	6	curative	CTX, RTX	burst abdomen	3
**3**	46	m	NOS	peritoneal cavity	2	1	palliative	none	pneumonia with fatal respiratory insufficiency, impaired wound healing	0
**4**	48	f	NOS	uterus	4	10	palliative	CTX, RTX	none	68
**5**	88	m	NOS	peritoneal cavity	2	1	palliative	none	gastrojejunal stenosis	12
**6**	18	f	DSRCT	peritoneal cavity/ovaries	0	1	curative	CTX	impaired wound healing	25
**7**	18	f	DSRCT	peritoneal cavity	20	3	curative	CTX , Hyperthermia	impaired wound healing	4
**8**	18	m	DSRCT	peritoneal cavity	0	4	curative	CTX , Hyperthermia	kidney failure, hemorrhagic shock and gastric perforation	22
**9**	24	m	DSRCT	peritoneal cavity, liver	4	3	curative	CTX , Hyperthermia	impaired wound healing	20
**10**	36	f	DLS	retroperitoneal space	2	1	palliative	CTX, Hyperthermia	none	6
**11**	84	m	DLS	pelvic region	0	1	palliative	none	fatal multiorgan failure	1
**12**	65	f	DLS	peritoneal cavity	19	4	palliative	CTX, RTX, Hyperthermia	urinary sepsis	2
**13**	44	m	MLS	gluteal region	46	7	curative	CTX, RTX, Hyperthermia	impaired wound healing	30
**14**	64	m	MLS	thigh	35	3	palliative	CTX, RTX	none	15
**15**	44	f	LMS	vaginal stump	0	4	curative	CTX , Hyperthermia	none	207

**16**	58	f	LMS	uterus	17	8	curative	CTX	none	82
**17**	24	f	MPNST	pelvic region	13	4	curative	CTX, RTX, Hyperthermia	pleuraempyema and hematothorax	2
**18**	68	m	SFT	pelvic region	27	2	curative	none	anastomotic leak, respiratory insufficiency	43
**19**	26	m	OS	tibia	34	3	palliative	CTX	pneumothorax after chest tube removal	9

Peritoneal sarcomatosis (PS), pleomorphic sarcomas – not otherwise specified (NOS), Desmoplastic Small Round Cell Tumors (DSRCT), Dedifferentiated Liposarcoma (DLS), Myxoid Liposarcoma (MLS), Leiomyosarcoma (LMS), Malignant Peripheral Nerve Sheath Tumor (MPNST), Solitary fibrous tumor (SFT), Osteosarcoma (OS), Chemotherapy (CTX), Radiotherapy (RTX), Female (f), Male (m)
